# Association of Delta-6-Desaturase Expression with Aggressiveness of Cancer, Diabetes Mellitus, and Multiple Sclerosis: A Narrative Review

**DOI:** 10.31557/APJCP.2019.20.4.1005

**Published:** 2019

**Authors:** Zhila Arshad, Soheila Rezapour-Firouzi, Meysam Ebrahimifar, Alireza Mosavi Jarrahi, Mahshid Mohammadian

**Affiliations:** 1 *Department of Pathology of Anatomy, School of medicine, Baku University of Medical Sciences, Baku, Azerbaijan,*; 2 *Cellular and Molecular Research Center, Cellular and Molecular Medicine Institute, *; 5 *Department of Biochemistry, School of Medicine, Urmia University of Medical Sciences, Urmia, *; 3 *Department of Toxicology, Faculty of Pharmacy, Islamic Azad University, Shahreza Branch, Shahreza, *; 4 *Department of Social Medicine, Medical School, Shahid Beheshti University of Medical Sciences, Tehran, Iran. *

**Keywords:** Cancer, Delta-6, Desaturase, Diabetes Mellitus, mTOR, Multiple sclerosis

## Abstract

**Background:**

The phosphatidylinositol 3-kinase/ protein kinase B /mammalian target of rapamycin (PI3K/Akt/mTOR) signaling regulates multiple cellular processes and organizes cell proliferation, survival, and differentiation with the available nutrients, in particular, fatty acids. Polyunsaturated fatty acids (PUFAs) are cytotoxic to cancer cells and play a critical role in the treatment of multiple sclerosis (MS) and diabetes mellitus (DM). PUFAs are produced in the body by desaturases and elongases from dietary essential fatty acids (EFAs), primarily involving delta-6-desaturase (D6D). D6D is a rate-limiting enzyme for maintaining many aspects of lipid homeostasis and normal health. D6D is important to recognize the mechanisms that regulate the expression of this enzyme in humans. A lower level of D6D was seen in breast tumors compared to normal tissues. Interestingly, the elevated serum level of D6D was seen in MS and DM, which explains the critical role of D6D in inflammatory diseases.

**Methods:**

We searched databases of PubMed, Web of Science (WOS), Google Scholar, Scopus and related studies by predefined eligibility criteria. We assessed their quality and extracted data.

**Results:**

Regarding the mTOR signaling pathway, there is remarkable contributions of many inflammatory diseases to attention to common metabolic pathways are depicted. Of course, we need to have the insights into each disorder and their pathological process. The first step in balancing the intake of EFAs is to prevent the disruption of metabolism and expression of the D6D enzyme.

**Conclusions:**

The ω6 and ω3 pathways are two major pathways in the biosynthesis of PUFAs. In both of these, D6D is a vital bifunctional enzyme desaturating linoleic acid or alpha-linolenic acid. Therefore, if ω6 and ω3 EFAs are given together in a ratio of 2: 1, the D6D expression will be down-regulated and normalized.

## Introduction

The mammalian or mechanistic target of rapamycin (mTOR) signaling in mammals is an intracellular kinase that regulates cell growth and development in response to growth factors, nutrients (amino acids, glucose, and fatty acids), cytokines and hormones such as insulin (Tremblay et al., 2001; Hands et al., 2009; Zoncu et al., 2011; Cornu et al., 2013). 

More recent studies have suggested that two mTOR complexes; namely, mTORC1 and mTORC2 are involved in the management of polyunsaturated fatty acids (PUFAs). Further, the compelling reasons have shown that mTOR dysregulation is explained by the progress of autoimmune diseases, including cancer cells, diabetes mellitus (DM), and multiple sclerosis (MS) (Blagosklonny, 2013; Giacoppo et al., 2017)

Cancer cells often use mTOR as a mechanism to increase their capacity to grow. The important roles of mTOR signaling delve into cell migration, inflammation, and regulation of transcription factors associated with cancer progression (Murray et al., 2018). The role of fatty acids in cancer risk and development is becoming more and more accepted, especially in terms of dietary intake of essential fatty acids (EFAs), which may be related to their special effects on the inflammatory processes (Dasilva et al., 2015). PUFAs suppress the expression of target genes as well as those involved in desaturation and elongation of fatty acids. Desaturases (FADS1, FADS2) and elongases (Elovl2, Elovl4, and Elovl5) are involved in PUFA synthesis. PUFAs are feedback inhibitors of their own synthesis (Jump et al., 2013). PUFAs should be taken up in the diet. They are the major components of phospholipids (PLs) structured by cell membranes that share the physical state and functions (Henderson, 1987). Environmental alterations are shown as important factors in the development of many diseases. The development of alternative treatments of DM and insulin resistance (IR) has been extensively studied in the last decades. Mistreatment of tuberculosis (TB) vaccination (Al-Attiyah et al., 2009), alteration of gut microbiota (Caricilli et al., 2013), intervention effects through phosphatidylinositol 3-kinase/ protein kinase B /mammalian target of rapamycin (PI3K/Akt/mTOR) signaling pathway (Blagosklonny, 2013) and the inhibition of delta-6-desaturase (D6D) enzyme activity by 2,2-diphenyl-5-(4-[[(1 E)-pyridin-3-yl-methylidene]amino]piperazin-1-yl) pentanenitrile (SC-26196), an inhibitor of D6D, are used to treat DM (Obukowicz et al., 1998). Despite the fact that the PI3K/Akt/mTOR signaling is involved in the management of PUFA, an inhibitor of D6D is used due to PUFA dysregulation. In addition, in the case of MS, EFAs are critical during the active period of myelination. If EFAs are not available in this period or are metabolically blocked, dysregulation of myelin synthesis may occur, including amyelination, dysmyelination, or demyelination (Auestad, 2000; Salvati et al., 2000). The mechanisms underlying disturbance in EFA metabolism of autoimmune diseases and abnormalities of D6D were reviewed in the current investigation. Surprisingly, in most of the autoimmune and inflammatory diseases, the excessive activity and expression of the D6D have been proven, although current treatments, regardless of the cause of increased activity, have focused on preventing its expression by mistreatment, which has been ineffective up to now. In this article, the role of the D6D enzyme in cellular metabolism and its natural regulation methods was discussed, in order to assist the management of intracellular mechanisms rather than the use of incorrect therapies.

*Role of PUFAs in Cell Membrane Fluidity*

Cell membranes mark the boundaries of live cells and their organelles and play a critical role in cellular connections and functions (Cooper et al., 2009). Cellular membranes are dynamic structures in which lipids are continuously added, removed, or modified to membrane type (Quinn et al., 1989). Consequently, membrane fluidity alterations, throughout cellular development, will be a fair mark for the correct performance of the cell wall. The fluidity of the cell membrane is involved in distinction functions, including permeability, deformability, transport of glucose, ions, or oxygen, and membrane-related enzymes (Knowles et al., 1976; Candiloros et al., 1995; Barclay, 2006). Cell membrane fluidity (CMF) may be a parameter describing the free movement of protein and lipid constituents at cell membrane intervals (Schroeder et al., 1976). In healthy tissue, CMF is at an optimum state which is vital for the mechanical behavior of the membrane. CMF is significantly affected by the cholesterol content, the fatty acids (FAs) constitution, and lipid-protein interactions (Shinitzky, 1984). Among the many components of the cell membranes are phospholipids (PLs) that contain FAs. A phospholipid is made of a saturated fatty acid (SFA), includes a different structure, and is a smaller amount of fluid than one that comprises an EFA (Yehuda et al., 2005). Additionally, an increase in the cholesterol/PL ratio reflects a decrease in fluidity (Shinitzky et al., 1978). Higher membrane PUFAs levels are associated with increased CMF. Given that diets deficient in EFAs is associated with CMF-influenced diseases (Rivers et al., 1981). CMF can be a vital indicator of cellular function maintenance. Alterations in CMF are seen in several inflammatory diseases.

*Maintenance of CMF in cancer, MS and DM*

Dietary fat is an essential nutrient and an important source for the EFAs, linoleic acid (LA 18:2n-ɷ6) and alpha-linolenic acid (ALA 18:3n-ɷ3), contributing to the appropriate growth and development. The development of colorectal, breast, prostate, endometrial and ovarian cancers is associated with the type and quality of fat, suggesting its underlying role. Tumor growth is the disruption of the homoeostatic balance, which regulates cell differentiation, proliferation, and apoptosis and it is related to altered lipid metabolism (Abel et al., 2014). Predominantly, changes in cell membrane FA metabolism related to ω6 and ω3-PUFA, are associated with alterations in membrane structure, function, cellular oxidative status, the activity of enzymes, and signaling pathways (Abel et al., 2014). Therefore, the normalization of CMF in these diseases may have therapeutic effects (Schroeder et al., 1976; Dobretsov et al., 1977) due to the availability of cell membrane receptors (Knazek et al., 1979) such as glucose transporter 4 (GLUT4) for glucose uptake in healthy tissue and inducing events occurring during the cell cycle (Lai et al., 1980). Finally, CMF could be a parameter for the different effects of the various ω6 / ω3-PUFAs ratios (Yehuda et al., 2000; Yehuda, 2003). EFAs determine CMF in neurons and manage the physiological functions of the brain. EFAs are additionally concerned with the synthesis and functioning of immune system molecules and brain neurotransmitters (Yehuda et al., 2005). Several studies have shown a relationship between mortality factor and dietary fat in patients with MS (Esparza et al., 1995). In addition, abnormalities of PUFAs synthesis could also be involved in MS (Ghadirian et al., 1998). If EFA deficiency occurs during the stages of life, there can be a significant delay in myelin synthesis, accompanied by impaired learning, vision, motor, and more additive abnormalities (Stockard et al., 2000). These abnormalities are associated with significant evidence of the elevations of pro-inflammatory eicosanoids like prostaglandin (PG)E2 (Horrobin et al., 1999). There is evidence that ω3-PUFAs can suppress elevation of pro-inflammatory eicosanoids like PGE2 and interferon (IFN)-γ production in patients with MS (Gallai et al., 1995). Low levels of each ω3-PUFAs and ω6-PUFAs in each plasma and red cells are found in depression. The ω3-PUFAs depletion is consistently bigger than the ω6-PUFAs depletion, resulting in elevations of the ω6/ω3, AA (arachidonic acid)/EPA (eicosapentaenoic acid), and AA/DHA (docosahexaenoic acid) ratios. These abnormalities are associated with significant elevation of pro-inflammatory eicosanoids like PGE2 (Horrobin et al.,1999). Current studies have shown dramatically reduced levels of ω3 and ω6-PUFAs in plasma, red blood cells, and adipose tissue of MS patients, and in a period of time, deficiency of PUFAs within the diet may also be a risk factor to develop MS (Holman et al.,1989). 

It has been shown that IR in non-insulin-dependent diabetes mellitus (non-IDDM) may be associated with changes in cell membrane properties (Tong et al., 1995). A number of clinical and metabolic abnormalities have been reported in diabetic nephropathy (DN) related to lipid bilayer modulation of cell membrane fluidity (Jones et al., 1998). Erythrocyte membranes isolated from diabetic patients have been shown to be scientifically fluidized in cell membranes, regardless of the type of diabetes or the presence of complications that could play a role in damaged cell functions in diabetes, which is controlled by the cell membrane (Waczulikova et al., 2000). Excess SFAs (by dietary or conversion of sugars into saturated fats by lipogenesis) results in rigid cellular membranes that in turn impair insulin signaling, glucose uptake, and blood circulation, making a malignant cycle that contributes to the development of obvious type 2 diabetes. Low membrane fluidity is an important component of diabetes pathophysiology (Pilon, 2016). A shift from PUFAs to the SFAs chains of membrane PLs, results in a more tight packing of PLs, decreasing the capacity for GLUT4 glucose transport (Garvey et al., 1998). For example, in type-2 diabetes, the concomitant increase in erythrocyte membrane stiffness can reduce the microcirculatory flow and cause tissue hypoxia and insufficient tissue nutrition, and lipid molecules can affect glucose transport by insulin-independent GLUTs and in turn glucose efficiency (Weijers, 2012). It has been shown that a significant decrease in erythrocyte membrane fluidity in patients with type 2 diabetes is dependent on the presence of SFAs and PUFAs (Mc Murchie et al.,1979; Rabini et al.,1993; Emam et al., 2008). Insulin sensitivity with the ratio of PUFAs/SFAs in membrane PLs is consistent with the notion that the amount of long-chain polyunsaturated fatty acids (Lc-PUFAs) in PLs is absolutely related to insulin sensitivity in healthy individuals (Borkman et al., 1993). Compared to healthy individuals, type 2 diabetic patients showed a significantly higher proportion of palmitate (C16:0-SFA) components in erythrocyte and leukocyte membranes and plasma samples (Bakan; 2006). Thus, development of each of cancer, DM and MS is accompanies dramatic alteration in the intake of dietary fatty acids in sufferers.

*Normalization of D6D activity and CMF by balancing ɷ*_6_ */ɷ*_3_ *-PUFAs*

**Figure 1:**
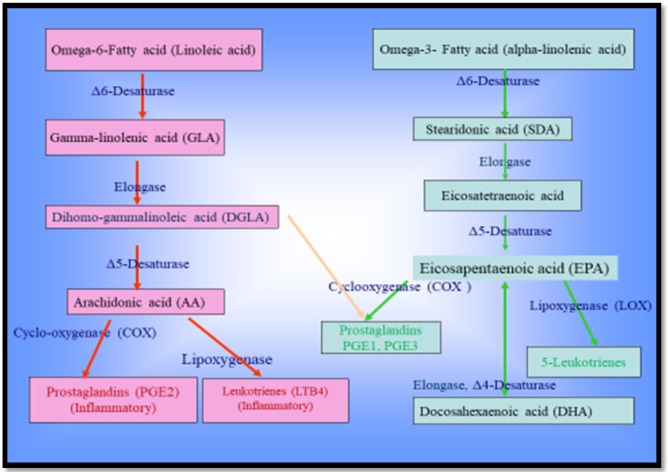
Metabolic Pathway of Polyunsaturated Fatty Acids

PUFAs are formed from LA and ALA, as the EFAs, by a series of elongation and desaturation reactions are mediated by elongases (Elovl2, Elovl4, Elovl5) and desaturases (D5D, D6D) (Beck, 2008; Miyazaki et al., 2008; Sul et al., 2008) (Figure 1.).

LA and ALA have an impression on the neuronal CMF. They are able to reduce the cholesterol level in the neuronal membrane, which can reduce CMF, and then make it hard for the cell to hold its best functions and increase the cell condition to tissue damage (Manku et al., 1984). CMF may also be implicated in the changes associated with the aging process. Age-related lowering of D6D activity will reduce anti-inflammatory eicosanoids synthesis such as PGE1 (Horrobin, 1981), which would be expected to increase CMF (Kury et al., 1974). D6D is the first enzyme of the sequence forming the gamma-linolenic acid (GLA) and stearidonic acid (STA or SDA) by adding a double link to LA and ALA, respectively. It is also responsible for the synthesis of ω6-AA and increased D6D activity can lead to enhanced ω6-AA production (He et al., 2012). There are strong positive correlations between serum PUFAs precursor/product ratios reflecting systemic D6D activity (e.g.,LA/AA) incidence of type 2 diabetes (Hodge et al., 2007; Kroger et al., 2011), and the development of metabolic syndrome (Vessby, 2003; Warensjo et al., 2005). Altogether, if ω6 and ω3 EFAs be given together with balance in ratio, GLA is rapidly converted to DGLA by elongase enzyme (Nakamura et al., 2004). From the DGLA, the PGE1 arises through the cyclooxygenase (COX1) enzyme, which has anti-inflammatory potency and modulates with a negative feedback mechanism of AA release in a free form from the membranous deposits (Borgeat et al.,1985) (Figure 1).

Therefore, the disturbance in the ratio of intake ω6 and ω3-EFAs in favor of ω6-FAs lead to increased production of ω6-AA, PGE2, leukotriene B4 (LTB4) and inflammation in cancer and allergy (Yu et al., 1998; Arshad et al., 2018). D6D activity is upregulated during inflammatory status in melanoma and lung tumor growth and suppressing D6D activity by balancing the ratio of intake of ω6 and ω3-EFAs (Rezapour-Firouzi et al., 2013a; Rezapour-Firouzi et al., 2013b; Rezapour-Firouzi et al., 2013c; Rezapour-Firouzi et al., 2013d) can reduce tumor growth. Consequently, the content of AA and AA-derived tumor-promoting metabolites is substantially reduced (He et al., 2012), without a need for treating a specific D6D inhibitor. As a result, angiogenesis and inflammatory status are also reduced. In mammals, the conversion of LA to GLA is slow, especially during stress, aging or diseases (hypertension, diabetes, etc.). If the conversion from LA to GLA is impaired by disturbing the enzyme D6D, dietary supplementation with GLA can help improve the situation. Moreover, excessive consumption of GLA metabolites: high rates of cell division, inflammatory and antiviral reaction and trauma. This is why the key deficits are to be in the production of GLA, dihomo-gamma-linolenic acid (DGLA), and PGE1 (Horrobin, 1990; Horrobin, 1992). 

Further, there is a positive notable role for D6D in insulin resistance and diabetes. Mechanisms responsible for increased D6D activity are imbalances of ɷ6/ɷ3-PUFAs; consequently, inhibition of D6D expression by SC-26196 (Obukowicz et al., 1998), can not be a proper therapeutic approache. The balance in the ratio of ɷ6-PUFAs/ ɷ3-PUFAs suppresses the expression of target genes, including desaturases and elongases; in addition PUFAs are feedback inhibitors of their own synthesis (Jump et al., 2013). Therefore, increased D6D would be expected to result in PUFA precursor/product imbalance in obese, diabetes and hyper insulinemia individuals as a hallmark. The activity of D6D is impaired by viral infection, aging, high blood pressure, high alcohol intake, high level cholesterol, stress-related hormones, radiation, nutritional factors (deficiencies of Zn+, Mg+, vitamins: C, B5, B6, B3 and high level of trans fatty acid), diabetes (Horrobin, 1990; Horrobin, 1992), and genetic deficiency (inactive D5D and D6D enzymes) (Bates, 1988), which can reduced DGLA production and then PGE1. Their relative deficiency may lose the negative feedback mechanism performed on AA, leading to its continued release from deposits and making it available for the formation of preferred high pro-inflammatory eicosanoids PGE2. The PGE2 is drawn from the AA through the COX2 pathway, while in normal people, the free AA concentration is low (Ruzicka et al., 1986). Moreover, alteration in exposure to PUFAs during pregnancy can affect the development of autoimmune diseases (Pike et al., 2012) including MS, type 1 DM, and atopic diseases. It has been indicated that experiments in the utilization of EFA in cancer modulation exist regarding intake and effect on cell structure and biochemical interactions within the cell in the prevention of cancer development. A diet containing high levels of ω6-FA, such as corn oil, has been shown to increase the development of tumors (Reddy, 1994). As a result, utilizing dietary PUFA in a specific ω6/ω3 ratio may be an essential chemo preventive tool in modifying the growth characteristics of cancer cells, since they are the major components of PLs structured by cell membranes that share the physical state and functions (Abel et al., 2014). Therefore, dramatic changes in the cellular lipid composition in favor of ω6-FA can disturb the structural and functional properties of membranes, thereby altering the growth features of neoplastic cells (Abel et al., 2014), including the increased intake of processed foods rich in SFA and trans FA and an imbalance change in EFA intake (Malhotra, 2013). It seems that the ideal ratio of ω6/ ω3 fatty acids should be 2.3:1 and even lower (1:1); this ratio needs to be reached because of these two groups of EFAs, complete distinct and complementary functions (Roncone et al., 2010). Present studies have estimated that the ω6/ω3 PUFAs ratio in developed nations are as high as 25:1 advising people that it should be too much lower (Delaleu et al., 2008). Altogether, if ω6 and ω3 EFAs be given together in 2.3:1 ratio, the D6D expression would be down-regulated; and it is not necessary to use SC-26196 to inhibit D6D expression in DM (Obukowicz et al., 1998) or in cancer (He et al., 2012). In a previous study, the intervention of a mixture of hempseed oil (HSO) and evening primrose oil (EPO) with ω6/ω3 EFAs (with a ratio of 2.3:1) in relapsing-remitting (RR) MS patients could down-regulate D6D, and phospholipases A2(PLA2) expression. (Rezapour-Firouzi et al., 2015, Rezapour-Firouzi, 2017).

*Role of Phospholipases A2 in Cancer, MS and DM*

Bioactive lipids are generated by hydrolysis of membrane lipids, mainly by phospholipases giving rise to FAs and lysophospholipids (lyso-PLs) that either directly exert their function or are further converted to active mediators and regulate several basic cell responses (Huwiler et al., 2009). Functional reactions of PUFAs in membrane PLs is severely controlled by PLA2 and acyltransferases, which are referred to as the “deacylation-reacylation cycle” (Sun et al., 2004). The important role of PLA2 in cellular death and tissue damage is revealed that follows through necrosis or apoptosis (Caro et al., 2006). Furthermore, the secretory PLA2 (sPLA2) involves many inflammatory conditions and implicates most of the membranes in any organ of the body (Yedgar et al., 2006). The PLA2 super family mobilizes free fatty acids (FFAs) by hydrolyzing of PLs and releasing lyso-PLs in the blood. Actually, inhibition of PLA2 would result in reduced levels of AA, which is necessary for pro-inflammatory eicosanoids production (Obukowicz et al.,1998). This is not possible unless sufficient EFAs are available in the diet to control any EFA metabolism by elongases and desaturases (Henderson, 1987). A number of FFAs will mediate inflammation and hallmarks of autoimmune diseases, such as AA (Masuda et al., 2005). Increase of PLA2 activity leads to accelerated membrane PLs hydrolysis and, in turn, increased plasma membrane permeability and cell lysis (Caro et al., 2006). The strategy for controlling inflammatory lipid mediator production is to protect the cell membrane against sPLA2 (Yedgar et al., 2006). To evaluate the PLA2-COX coupling result in pro-inflammatory eicosanoids PGE2 generation, sPLA2 was induced after cytokine stimulation, with IFN-γ, the hallmark of Thelper (Th) 1 cells, exhibiting a potent effect than interleukin (IL)-1β or tumor necrosis factor (TNF)-α (Masuda et al., 2005). PLA2 plays a greater role in the clearance of myelin by macrophages and a critical role in the progression of MS (Lopez-Vales et al., 2008; Kalyvas et al., 2009). Regardless of immunomodulation therapy, mean levels of sPLA2 were increased 6-fold in the urine of MS patients in relapses or in the active phase of disease and 4-fold in patients in remission (Cunningham et al., 2006). Alterations in the proportions of various FA classes cause a decrease in CMF in membranes with an increase in PLA2 classes in the serum of MS patients. With respect to gained evidence, the disturbance in the structure of PLs and the dysregulation of lipid metabolism in the myelin and sub cellular membrane, such as the inner mitochondrial membrane (IMM), along with a wide variety of enzymatic activities can contribute to the acute apoptosis of oligodendrocytes and neurons of the central nervous system (CNS) in MS development (Rezapour-Firouzi, 2017). PLA2s have been implicated in the pathology of a number of neurodegenerative diseases. In particular, in the CNS, sPLA2 mRNA is expressed in response to pro-inflammatory cytokines TNF-α, IL-1β, and IFN-γ (Sun et al., 2004). In human cells, IL-1β and TNF-α mediated the increase in PLA2 activity and the release of AA (Chenevier-Gobeaux et al., 2007), and its link with COX-2 and PGE2 in exacerbating inflammatory events under pathological conditions.

Surprisingly, PLA2 may be implicated in the pathogenesis of IR, leading to diabetes through inflammatory modulation. The tissue damage of DM and MS patients involve in lipoprotein-associated PLA2 (Lp-PLA2) with pro-inflammatory activity and it is related to the generation of significant amounts of lysophosphatidylcholines (lyso-PC) and AA from the degradation of phosphatidylcholine (PC), leading to inflammatory cell activation (MacPhee et al., 1999; Sternberg et al., 2012). Lp-PLA2 may be a member of the intracellular and sPLA family secreted by activated macrophages (Lerman et al., 2008; Vittos et al., 2012). Although generation of lyso-PLs and FFAs by LP-PLA2 contribute to its pro-inflammatory status, diabetic patients may be involved with some nutritional factors contributing to the regulation of this enzyme (Cheraghi et al., 2015). 

Several sPLA2s have been associated with the initiation and progression of certain types of various cancers, including lung, breast, prostate, colon, and gastric cancers. The expression of some sPLA2s is up regulated in various tumor tissues. The role of sPLA2s in cancer has been generally associated with their enzymatic activity and the ability to participate in the release of an effective biologically active lipid mediator, exactly AA-derived eicosanoids such as PGE2, promoting tumor igenesis by stimulating cell proliferation and cell survival through abolishing apoptosis and increasing local inflammation and angiogenesis (Brglez et al., 2014). PLA2 has also been identified as a potential target of cancer therapy (Quach et al., 2014). 

*Role of mTOR Pathway in Management of PUFAs and vice versa*

**Figure 2:**
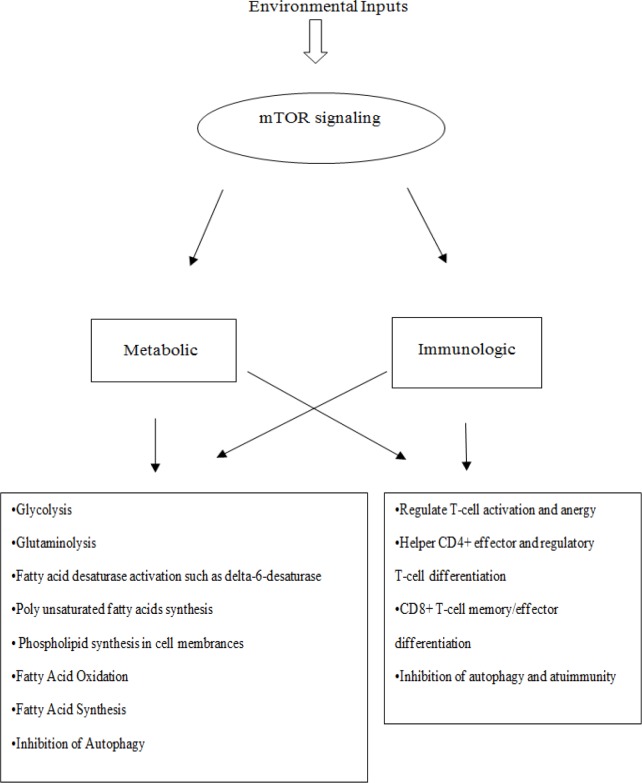
Metabolic and immunologic pathway underling mTOR signaling

The mTOR kinase that is activated by nutritional signals plays a fundamental role in regulating fatty acids and metabolism in response to nutrients. The mTOR kinase are two large protein complexes known as mTOR complex 1 (mTORC1) and mTOR complex 2 (mTORC2). Following their activation, these complexes facilitate the accumulation of triglycerides by promoting a dipogenesis and lipogenesis (Caron et al., 2015). The mTOR signaling pathway plays a crucial role in dictating the T cell fate through the interaction and balance of two mTOR-containing complexes (Kim et al., 2002; Zoncu et al., 2011). Although the control of lipogenesis was mainly reported in relation to mTORC1, recent studies have indicated that mTORC2 also plays a critical role in modulating lipid synthesis (Hagiwara et al., 2012; Yuan et al., 2012). The mTORC1 may be critical for the long-term regulation of lipid homeostasis. Reduced mTORC1 activity increases lipolysis and decreases mitochondrial oxidization of FFA (Chakrabarti et al., 2015). The mTOR signaling, which plays a central role in the regulation of mRNA translation in mammalian cells (Redig et al., 2011), has been reported to activate the transcription factor, SREBP (Sterol Regulatory Element Binding Protein) (Brown et al., 2007), which in turn activates acetyl-CoA carboxylase (ACC) (Peng et al., 2002), fatty acid desaturase (FASD) (Mauvoisin et al., 2007), stearoyl-CoA desaturase (SCD) (Lamming et al., 2012), and enzymes involved in lipogenesis (Brown et al., 2007) (Figure 2). 

The anabolic role of mTOR is evident in the regulation of lipogenesis as mTORC1 activates the transcription factor SREBP, the main transcription factor that controls FAs synthesis (Peng et al., 2002; Mauvoisin et al., 2007; Porstmann et al., 2008), and SCD, a vital enzyme in FAs metabolism required for the formation of double bond (Kim et al., 2004). The mTORC1 also promotes the expression and activation of peroxisome proliferator-activated receptor γ (PPAR-γ), the principal regulator of adipogenesis (Zhang et al., 2009). PPAR plays critical roles in the management of cell growth, differentiation, and metabolism (protein, carbohydrate, and lipid) (Dunning et al., 2014). The mTORC1-PPARγ pathway is important for the FAs uptake plan in activated CD4+ T cells. This pathway is required for the full activation and rapid proliferation of naive and memory CD4+ T cells. It means that FA metabolism controls the full activation of CD4+ T cells. FAs are known to be essential metabolites for maintaining cell activation, proliferation, and functioning in rapidly proliferating cells (Angela et al., 2016). It appears that ɷ3-PUFAs have an effect in the first year of life management of the immune system (Calder et al., 2010). FAs have a significant impact on mTORC1 regulation (Yasuda et al., 2014). While mTORC1 is activated by the SFAs such as palmitate, via enhancing the translocation of mTORC1 to the lysosome, ɷ3-LC-PUFAs such as EPA inhibit SFA-induced translocation of mTORC1 to the lysosome; subsequently, induce its activation (Redig et al., 2011). ɷ3-LC-PUFAs-mediated function mitigates mTORC1 activity and may increase the maturation and functioning of T regulatory (Treg) cells (Zivkovic et al., 2011), which is similar to the administration of rapamycin (RAPA; an inhibitor of mTOR) in the induction of tolerance through expansion of Treg cells (Wang et al., 2013). Therefore, the quality of fat, including reduced ratio of the ω6 /ω3 fatty acids or intake increase of ɷ3-PUFAs, as well as administration of rapamycin (RAPA), can ameliorate autoimmune diseases by inhibiting T-helpers: Th1/Th2/Th17 cells and up-regulating of Treg cells via the control of mTOR signaling in autoimmune diseases (Sakaguchi, 2004; Yuan et al., 2015; Rezapour-Firouzi, 2017). RAPA, or sirolimus, is a macrocyclic triene antibiotic and a natural product produced by fermentation of *Streptomyces hygroscopic* strain displaying antitumor and immunosuppressive activities; it has been generally used for the prevention of clinical allograft rejection and for treating some type of cancer and autoimmune diseases (Kelly et al., 1997). RAPA can prevent the induction and the progression of the relapsing–remitting experimental autoimmune encephalomyelitis (RR-EAE), a commonly used animal model for studying RR-MS pathology (Esposito et al., 2010). Of course, the adverse side effects of RAPA such as anemia, thrombocytopenia, nausea, headache, fever, urinary tract infection and interstitial pneumonitis have limited the usefulness of this drug (Merkel et al., 2006). However, the reduced proportions of ω6 / ω3 fatty acids or intake increase of ω3-PUFAs are without side effect and complications.

In summary, the survey of biochemical and immunological factors in diseases of allergy (as we have described in Arshad et al., (2018), cancer, DM, and MS has not only provided insights into the pathogenesis of diseases but also lead to the development of potentially new therapeutic targets and validated approved therapies. The similarities and differences between the biochemical and immunological factors involved in these diseases are summarized in (Table 1).

**Table 1:** The Biochemical and Immunological Factorsinvolved in Diseases of Cancer, Allergy, Diabetes Mellitus, and Multiple Sclerosis

	CMF and LA/ALA balance	PUFAs P/P balance	mTORC1 mediated	COX/ PGE2/ Th1	mTORC2-mediated	LOX/ LTB4/ Th2	Therapy with RAPA	D6D activity	sPLA2
Cancer	reduced	reduced	_	_	elevated	elevated	Yes	abnormality	elevated
* Allergy	reduced	reduced	_	_	elevated	elevated	No	abnormality	elevated
Diabetes Mellitus	reduced	reduced	elevated	elevated	_	_	controversial	abnormality	elevated
Multiple Sclerosis	reduced	reduced	elevated	elevated	_	_	in animal model	abnormality	elevated

LA/ALA, linoleic acid/linolenic acid; PUFAs - P/P, poly unsaturated fatty acids-precursor/product; mTORC, mammalian target of rapamycin complex; COX, cyclooxygenase; PGE2, prostaglandin E2; LOX, lipoxygenase; LTB4, leukotriene B4; Th, T helper; RAPA, rapamycin D6D: delta-6-desaturase; sPLA2, secretory PLA2.*, Refer to the article (Arshad et al., 2018).

Table 1 shows that reduced CMF, decreased EFAs balance intake in favor of LA and imbalance in PUFAs precursors/products are common features of these chronic diseases that are presented by immunological abnormalities. This means that imbalance in LA/ALA intake and biochemical reactions results in imbalance in immunological reactions.

## Discussion

The obtained evidence has revealed that the inhibition of mTOR attenuates the differentiation of effector T cells, whereas complete inhibition of mTORC1 largely mitigates Th1 differentiation. However, it leaves Th2 differentiation complete, and selectively deletes SGK1 (downstream of mTORC2) by reducing Th2 differentiation while leaving Th1 differentiation intact (Kleinewietfeld et al., 2013; Wu et al., 2013). Extreme activation of phosphorylation of PI3K/AKT/mTOR signaling is one of the supreme common alterations in human cancers (Qi et al., 2017). Many studies have established dysregulation of the PI3K/AKT/mTOR pathway in tumors, and mainly in the biologic regulation of the breast cancer (Mohan et al., 2016), gastric cancer (Samuels, Wang et al., 2004), liver cancer (Grabinski et al., 2012), colorectal cancer (Francipane et al., 2014) and prostate cancer (Chang et al., 2015). In addition, allergic people have a high chance of getting cancer. Since the immune system tends to be on the Th2 immune cells in the cancers. Thus, the increase in PI3K/AKT/mTOR signaling activity is due to the creation of a series of cytokines in the immune system of Th2 cells in cancers and allergies (Arshad et al., 2018). In contrast, increasing the activity of PI3K/AKT/mTOR pathway in MS and DM is a therapeutic approach that is opposed to cancer and allergies. Although scientific evidence does not mention the role of this pathway in activating the pathway of Th2 immune cells, it seems that this route will advance the immune system towards Th2 immune cells. On the other hand, mTOR pathway is stimulated by environmental and nutritional factors, therefore, in people whose immune system is somehow directed towards Th2 or Th1 immune cells, the immune system can be exacerbated or weakened by environmental and nutritional factors. For example, eating allergic foods will exacerbate Th2 immune route in an allergic person or a cancer patient. This is the same concept that has been cited in detail in Traditional Iranian Medicine (TIM) for many years (Arshad et al., 2018). As it mentioned, mTORC2 is important for Th2 differentiation; whereas, mTORC1 is vital for Th1 and Th17 differentiation (Lee et al., 2010). Recent results have shown that the phosphorylation of PI3K/AKT/mTOR pathway or activation of mTORC2 is also a helpful target in the development of remyelinating therapies for demyelinating diseases (Narayanan et al., 2009). Recently, the exact role of mTOR in the modulation of glial functions has also been found. Previous studies support the notion that mTOR is involved in glial proinflammatory activation and it also controls the biosynthesis of lipids required for cells proliferation to generate cell membranes (Laplante et al., 2009). Reports have demonstrated the involvement of the PI3K/AKT/mTOR pathway in oligodendrocyte differentiation and during CNS myelination development (Flores et al., 2008). Some of the studies reported that activation of the PI3K/AKT/mTOR signaling is a pathway required for oligodendrocyte survival and axon myelination (Kumar et al., 2013).

On the other hand, in adipocytes and muscle cells, insulin activates the insulin receptor tyrosine kinase (IR), which subsequently activates the tyrosine phosphorylation of the insulin receptor substrate (IRS) proteins. Once activated, these IRSs activate phosphatidylinositide-3-kinase (PI3K), catalyzing the production of phosphatidylinositol-triphosphate (PIP3) from phosphatidylinositol-biphosphate (PIP2) (Hotamisligil et al., 1993; Bammens et al., 2004; Turnbaugh et al., 2006), triggering the phosphorylation of protein kinase B/Akt (PKB/Akt), and allowing the translocation of insulin-sensitive GLUT4 to the plasma membrane, and facilitating the uptake of glucose (Samuel et al., 2012). Studies on animal have demonstrated that insulin and its signaling cascade normally control cell growth, metabolism, and survival through activation of mitogen-activated protein kinases (MAPKs) and phosphotidylinositide-3-kinase (PI3K), of which activation of PI-3K- is associated with control of nutrient homeostasis and organ survival. Inactivation of Akt in different organs following hyperinsulinemia, metabolic inflammation, and over nutrition could offer the underlying mechanisms for metabolic syndrome in humans (Guo, 2014). The PI 3-kinase and its downstream target, Akt, promote insulin-induced movement of GLUT4 to the cell membrane, glucose uptake, glycolysis, glycogen synthesis, and protein synthesis (Kohn et al., 1996; Cong et al., 1997; Calera et al., 1998; Ueki et al., 1998; Foran et al., 1999; Hill et al., 1999; Wang et al., 1999). PI 3-kinase-Akt signaling promotes and regulates insulin action (Egawa et al., 1999). Therefore, the mTORC2 pathway is strongly implicated in the metabolic switch as a result of its activation upregulates the surface expression of the glucose transporter, GLUT1, through PI3K and protein kinase B (PKB also known as Akt) (Wieman et al., 2007). Akt signaling through mTORC2 also results in higher expression of amino acid and other nutrient transporters, such as the transferrin receptor (Zheng et al.,2007). Activation of PI3K and Akt plays a central role in metabolic regulation. Mice lacking Akt2 developed type 2 diabetes mellitus (Cho et al., 2001), and Akt2 mutation has also been described in patients with type 2 diabetes mellitus (George et al., 2004). It is notable is that long-term treatment with RAPA blocks mTORC2-mediated Akt phosphorylation/activation; therefore the use of RAPA for the treatment type 2 diabetes can be a clinical challenge (Sarbassov et al., 2005). Of note, with regard to mTORC2, is important for Th2 differentiation, so the deviation immune system toward secretion of cytokines of Th2 has therapeutic effects on MS and DM. For instance, they are essential for the emergence of Th2 cell subsets and the differentiation of gut B cells into immunoglobulin (Ig) A-producing plasma cells (Caricilli et al., 2013), or mistreatment of TB vaccination (Al-Attiyah et al., 2009), which related to the development of alternative treatments of DM and insulin resistance (IR), has been extensively studied in the last decades. The insulin signaling may also be impaired by altered secretion of cytokines and chemokines. For example, in type 2 diabetic patients, circulating T cells produce higher levels of IL-17 and IFN-γ, leading to a pro-inflammatory state (Jagannathan-Bogdan et al., 2011). Other cytokines, such as TNF-α and IL-6, are also related to insulin resistance. TNF-α protein is elevated is adipose and the inhibition of its expression leads to increased peripheral uptake of glucose (Hotamisligil et al., 1993; Federici et al., 2005; Monroy et al., 2009). IL-6 can also affect insulin signaling: its plasma concentration is inversely proportional to insulin sensitivity, which is hypothesized by IL-6-induced suppressors of cytokine signaling (SOCSs) expression. SOCSs usually suppress the effect of cytokines on insulin transduction steps, such as IRS-1 phosphorylation, PI3K or PKB activation (Kern et al., 2001; Senn et al., 2003). Administration of IL-12, a key cytokine which guides the development of Th1 CD4+ T cells, induces rapid onset of insulin-dependent diabetes mellitus (IDDM) in the nonobese diabetic (NOD) mice, but not in BALB/c mice (With tendency of immune cells toward Th2). IL-12 administration accelerates IDDM development in genetically susceptible NOD mice and this correlates with increased Th1 cytokine production by islet-infiltrating cells. These results hold implications for the pathogenesis and, possibly, for the treatment of IDDM and other Th1 cell-mediated autoimmune diseases (Trembleau et al., 1995). Conversely, the Th2-derived cytokines IL-4 and IL-10 appear to inhibit progression to IDDM in NOD mice (Rapoport et al., 1993; Pennline et al., 1994, Scott et al., 1994). DN is considered the main cause of end-stage kidney disease around the world. It has been shown that concentrations of pro-inflammatory cytokines, as IL-1, IL-6, IL-18, IL-33, IFN-γ and TNF-α actively participate in the development and progression of DN, and thus it seems that they are involved in pathogenesis, which is similar to MS. In addition, changes in the acquired immune response, especially the presence of the pro-inflammatory and effector nature of the cellular immune response profiles, in particular Th1 and Th17, as the imbalance between the interaction of cytokines and T regulatory cells, initiate the onset and progression of DN (Araujo et al., 2016), which is similar to MS. Patients with DN had a more evident Th1 profile characterized by increased IFN-γ, IL-2 and IL-12 and decreased Th2 cytokines IL-33 and IL-13, indicating that DN can be characterized by an increase in Th1 associated with suppression of Th2 response (Anand et al., 2014).

In the case of MS, similar DN alteration of the cytokine profiles have been documented within CNS tissue (Brosnan et al., 1995) and peripheral blood mononuclear cells (PBMCs) derived from MS patients (Imitola et al., 2005). Some of increased cytokines in MS include, IFN-γ (Balashov et al., 2000), TNF-α (Cannella et al., 1995), IL-8 (Nicoletti et al., 2001), IL-6 (Schonrock et al.,2000), and IL-17 (Miossec et al., 2009). In the majority of MS cases, several Th1 cytokines increase, while Th2 cytokines decrease. Given that several Th2 cytokines have been implicated in the survival of neurons and oligodendrocytes, therapies promoting a Th1 to Th2 cytokine shift are supported in MS (Imitola et al., 2005). 

However, the bias of the immune system toward Th2, which leads to an exacerbation of cancer and allergic diseases, or the bias of the immune system toward Th1 which is due to the intensification of MS and DM, the root cause of all these diseases is the imbalance in the intake of ω3 and ω6-EFAs of suitable source of fatty acids based on TIM, as was explained in (Arshad et al., 2018), is the condition for the prevention of inflammation and the regulation of metabolic and emotional processes. The equilibrium in the entry of ω3 and ω6-essential fatty acids in the PUFA metabolic cycle causes the balance in the function of desaturases and elongases in the process of desaturation and elongation PUFAs and the production metabolites, preventing the excessive desaturases enzymes expression by establishing mechanistic feedbacks for them.

In conclusion, there are substantial reasons to believe that the inhibition of inflammatory aspects such as elevation of delta-6-desaturase activity in cancer, allergy, multiple sclerosis, and diabetes mellitus can be reduced by the supplementation of essential fatty acids or gamma-linolenic acid. The supplementation should be provided from suitable sources based on TIM, as was explained in the previous study (Arshad et al., 2018), to increase the cell membrane fluidity and precursor/product balance of polyunsaturated fatty acids (ω3-PUFAs, ω6-PUFAs) as therapeutic targets.

## Authorship contribution

Study concepts/study design: all authors; Collection demographic data: all authors; manuscript drafting: Zhila Arshad; manuscript final version approval and manuscript editing: Soheila Rezapour-Firouzi, Mahshid Mohammadian and Meysam Ebrahimifar.

## Declaration of interest

All the authors declare that they have no conflict of interests.

## References

[B1] Abel S, Riedel S, GelderblomWC (2014). Dietary PUFA and cancer. Proc Nutr Soc.

[B2] Al-Attiyah RJ, Mustafa AS (2009). Mycobacterial antigen-induced T helper type 1 (Th1) and Th2 reactivity of peripheral blood mononuclear cells from diabetic and non-diabetic tuberculosis patients and Mycobacterium bovis bacilli Calmette-Guerin (BCG)-vaccinated healthy subjects. Clin Exp Immunol.

[B3] Amiri B, Ebrahimi Far M, Saffari Z (2016). Preparation, characterization and cytotoxicity of silibinin containing nanoniosomes in T47D human breast carcinoma cells. Asian Pac J Cancer Prev.

[B4] Anand G, Vasanthakumar R, Mohan V (2014). Increased IL-12 and decreased IL-33 serum levels are associated with increased Th1 and suppressed Th2 cytokine profile in patients with diabetic nephropathy (CURES-134). Int J Clin Exp Pathol.

[B5] Angela M, EndoY, Asou HK (2016). Fatty acid metabolic reprogramming via mTOR-mediated inductions of PPARgamma directs early activation of T cells. Nat Commun.

[B6] Araujo LS, da SilvaMV, da SilvaCA (2016). Cytokines and T helper cells in diabetic nephropathy pathogenesis. J Diabetes Mellitus.

[B7] Arshad Z, Rezapour-FirouziS, Mohammadian M (2018). The sources of essential fatty acids for allergic and cancer patients; a connection with insight into mammalian target of rapamycin: A narrative review. Asian Pac J Cancer Prev.

[B8] Auestad N (2000). Infant nutrition--brain development--disease in later life An introduction. Dev Neurosci.

[B9] Bakan E, Yildirim A, KurtulN (2006). Effects of type 2 diabetes mellitus on plasma fatty acid composition and cholesterol content of erythrocyte and leukocyte membranes. Acta Diabetol.

[B10] Balashov KE, ComabellaM, OhashiT (2000). Defective regulation of IFNgamma and IL-12 by endogenous IL-10 in progressive MS. Neurology.

[B11] Bammens B, EvenepoelP, VerbekeK (2004). Impairment of small intestinal protein assimilation in patients with end-stage renal disease: extending the malnutrition-inflammation-atherosclerosis concept. Am J Clin Nutr.

[B12] Barclay L, NghiemHT (2006). Fish oil supplements during pregnancy are safe and beneficial. Arch Dis Child Fetal Neonatal.

[B13] Bates CE (1988). Racially determined abnormal essential fatty acid and prostaglandin metabolism and food allergies linked to autoimmune, inflammatory, and psychiatric disorders among Coastal British Columbia Indians. Med Hypotheses.

[B14] Beck C, Vance JE,, Vance D (2008). Assembly and secretion of atherogenic lipoproteins. Biochemistry of lipids, lipoproteins and membranes.

[B15] Blagosklonny MV (2013). TOR-centric view on insulin resistance and diabetic complications: perspective for endocrinologists and gerontologists. Cell Death Dis.

[B16] Borgeat P, NadeauM, SalariH (1985). Leukotrienes: biosynthesis, metabolism, and analysis. Adv Lipid Res.

[B17] Borkman M, StorlienLH, PanDA (1993). The relation between insulin sensitivity and the fatty-acid composition of skeletal-muscle phospholipids. N Engl J Med.

[B18] Brglez V, Lambeau G, PetanT (2014). Secreted phospholipases A2 in cancer: diverse mechanisms of action. Biochimie.

[B19] Brosnan CF, CannellaB, Battistini L (1995). Cytokine localization in multiple sclerosis lesions: correlation with adhesion molecule expression and reactive nitrogen species. Neurology.

[B20] Brown NF, Stefanovic-RacicM, Sipula IJ (2007). The mammalian target of rapamycin regulates lipid metabolism in primary cultures of rat hepatocytes. Metabolism.

[B21] Calder PC, KremmydaLS, VlachavaM (2010). Is there a role for fatty acids in early life programming of the immune system?. Proc Nutr Soc.

[B22] Calera MR, MartinezC, LiuH (1998). Insulin increases the association of Akt-2 with Glut4-containing vesicles. J Biol Chem.

[B23] Candiloros H, MullerS, ZeghariN (1995). Decreased erythrocyte membrane fluidity in poorly controlled IDDMinfluence of ketone bodies. Diabetes Care.

[B24] Cannella B, Raine CS (1995). The adhesion molecule and cytokine profile of multiple sclerosis lesions. Ann Neurol.

[B25] Caricilli AM, Saad MJ (2013). The role of gut microbiota on insulin resistance. Nutrients.

[B26] Caro AA, CederbaumAI (2006). Role of cytochrome P450 in phospholipase A2- and arachidonic acid-mediated cytotoxicity. Free Radic Biol Med.

[B27] Caron A, Richard D, Laplante M (2015). The roles of mTOR complexes in lipid metabolism. Annu Rev Nutr.

[B28] Chakrabarti P, Kandror KV (2015). The role of mTOR in lipid homeostasis and diabetes progression. Curr Opin Endocrinol Diabetes Obes.

[B29] Chang L, GrahamPH, NiJ (2015). Targeting PI3K/Akt/mTOR signaling pathway in the treatment of prostate cancer radioresistance. Crit Rev Oncol Hematol.

[B30] Chenevier-Gobeaux C, SimonneauC, TherondP (2007). Implication of cytosolic phospholipase A2 (cPLA2) in the regulation of human synoviocyte NADPH oxidase (Nox2) activity. Life Sci.

[B31] Cheraghi A, MahmoudiM, JafarianK (2015). Comparison of serum LP-PLA2 level and some nutritional factors between well-controlled and poorly-controlled diabetic patients. Acta Med Iran.

[B32] Cho H, MuJ, KimJK (2001). Insulin resistance and a diabetes mellitus-like syndrome in mice lacking the protein kinase Akt2 (PKB beta). Science.

[B33] Cong LN, ChenH, LiY (1997). Physiological role of Akt in insulin-stimulated translocation of GLUT4 in transfected rat adipose cells. Mol Endocrinol.

[B34] CooperGM, Hausman RE (2009). The plasma membrane. The cell: A molecular approach.

[B35] Cornu M, AlbertV, Hall MN (2013). mTOR in aging, metabolism, and cancer. Curr Opin Genet Dev.

[B36] Cunningham TJ, YaoL, OetingerM (2006). Secreted phospholipase A2 activity in experimental autoimmune encephalomyelitis and multiple sclerosis. J Neuroinflammation.

[B37] Dasilva G, PazosM, Garcia-EgidoE (2015). Healthy effect of different proportions of marine omega-3 PUFAs EPA and DHA supplementation in Wistar rats: Lipidomic biomarkers of oxidative stress and inflammation. J Nutr Biochem.

[B38] Delaleu N, ImmervollH, CorneliusJ (2008). Biomarker profiles in serum and saliva of experimental Sjogren’s syndrome: associations with specific autoimmune manifestations. Arthritis Res Ther.

[B39] Dobretsov GE, BorschevskayaTA, Petrov VA (1977). The increase of phospholipid bilayer rigidity after lipid peroxidation. FEBS Lett.

[B40] Dunning KR, AnastasiMR, ZhangVJ (2014). Regulation of fatty acid oxidation in mouse cumulus-oocyte complexes during maturation and modulation by PPAR agonists. PLoS One.

[B41] Egawa K, SharmaPM, NakashimaN (1999). Membrane-targeted phosphatidylinositol 3-kinase mimics insulin actions and induces a state of cellular insulin resistance. J Biol Chem.

[B42] Emam SJ, NikzamirAR, Nakhjavani M (2008). Erythrocyte membrane fluidity in ageing, type 2 diabetes and stroke patients. Int J Endocrinol Metab.

[B43] Esparza ML, Sasaki S, KestelootH (1995). Nutrition, latitude, and multiple sclerosis mortality: an ecologic study. Am J Epidemiol.

[B44] Esposito M, RuffiniF, BelloneM (2010). Rapamycin inhibits relapsing experimental autoimmune encephalomyelitis by both effector and regulatory T cells modulation. J Neuroimmunol.

[B45] Federici M, HribalML, MenghiniR (2005). Timp3 deficiency in insulin receptor-haploinsufficient mice promotes diabetes and vascular inflammation via increased TNF-alpha. J Clin Invest.

[B46] Flores AI, NarayananSP, MorseEN (2008). Constitutively active Akt induces enhanced myelination in the CNS. J Neurosci.

[B47] Foran PG, FletcherLM, OateyPB (1999). Protein kinase B stimulates the translocation of GLUT4 but not GLUT1 or transferrin receptors in 3T3-L1 adipocytes by a pathway involving SNAP-23, synaptobrevin-2, and/or cellubrevin. J Biol Chem.

[B48] Francipane MG, Lagasse E (2014). mTOR pathway in colorectal cancer: an update. Oncotarget.

[B49] Gallai V, SarchielliP, TrequattriniA (1995). Cytokine secretion and eicosanoid production in the peripheral blood mononuclear cells of MS patients undergoing dietary supplementation with n-3 polyunsaturated fatty acids. J Neuroimmunol.

[B50] Garvey WT, Maianu L, Zhu JH (1998). Evidence for defects in the trafficking and translocation of GLUT4 glucose transporters in skeletal muscle as a cause of human insulin resistance. J Clin Invest.

[B51] George S, RochfordJJ, Wolfrum C (2004). A family with severe insulin resistance and diabetes due to a mutation in AKT2. Science.

[B52] Ghadirian P, JainM, DucicS (1998). Nutritional factors in the aetiology of multiple sclerosis: a case-control study in Montreal. Canada Int J Epidemiol.

[B53] Giacoppo S, Pollastro F, Grassi G (2017). Target regulation of PI3K/Akt/mTOR pathway by cannabidiol in treatment of experimental multiple sclerosis. Fitoterapia.

[B54] Grabinski N, Ewald F, Hofmann BT (2012). Combined targeting of AKT and mTOR synergistically inhibits proliferation of hepatocellular carcinoma cells. Mol Cancer.

[B55] Guo S (2014). Insulin signaling, resistance, and the metabolic syndrome: insights from mouse models into disease mechanisms. J Endocrinol.

[B56] Hagiwara A, CornuM, CybulskiN (2012). Hepatic mTORC2 activates glycolysis and lipogenesis through Akt, glucokinase, and SREBP1c. Cell Metab.

[B57] Hands SL, Proud CG, WyttenbachA (2009). mTOR’s role in ageing: protein synthesis or autophagy?. Aging (Albany NY).

[B58] He C, QuX, WanJ (2012). Inhibiting delta-6 desaturase activity suppresses tumor growth in mice. PLoS One.

[B59] Henderson WRJr (1987). Eicosanoids and lung inflammation. Am Rev Respir Dis.

[B60] Hill MM, ClarkSF, TuckerDF (1999). A role for protein kinase Bbeta/Akt2 in insulin-stimulated GLUT4 translocation in adipocytes. Mol Cell Biol.

[B61] Hodge AM, English DR, O’Dea K (2007). Plasma phospholipid and dietary fatty acids as predictors of type 2 diabetes: interpreting the role of linoleic acid. Am J Clin Nutr.

[B62] Holman RT, Johnson SB, Kokmen E (1989). Deficiencies of polyunsaturated fatty acids and replacement by nonessential fatty acids in plasma lipids in multiple sclerosis. Proc Natl Acad Sci U S A.

[B63] Horrobin DF (1981). Loss of delta-6-desaturase activity as a key factor in aging. Med Hypotheses.

[B64] Horrobin DF (1990). Gamma-linolenic acid: An intermediate in essential fatty acid metabolism with potential as an ethical pharmaceutical and as a food. Rev Contemp Pharmacother.

[B65] Horrobin DF (1992). Nutritional and medical importance of gamma-linolenic acid. Prog Lipid Res.

[B66] Horrobin DF, Bennett CN (1999). Depression and bipolar disorder: relationships to impaired fatty acid and phospholipid metabolism and to diabetes cardiovascular disease immunological abnormalities cancer ageing and osteoporosis Possible candidate genes. Prostaglandins Leukot Essent Fatty Acids.

[B67] Hotamisligil GS, ShargillNS, SpiegelmanBM (1993). Adipose expression of tumor necrosis factor-alpha: direct role in obesity-linked insulin resistance. Science.

[B68] Huwiler A, PfeilschifterJ (2009). Lipids as targets for novel anti-inflammatory therapies. Pharmacol Ther.

[B69] Imitola J, Chitnis T, Khoury SJ (2005). Cytokines in multiple sclerosis: from bench to bedside. Pharmacol Ther.

[B70] Jagannathan-Bogdan M, McDonnellME, ShinH (2011). Elevated proinflammatory cytokine production by a skewed T cell compartment requires monocytes and promotes inflammation in type 2 diabetes. J Immunol.

[B71] Jones SC, Thomas TH, MarshallSM (1998). Abnormal regulation of cell membrane fluidity in diabetic nephropathy. Diabetologia.

[B72] Jump DB, TripathyS, Depner CM (2013). Fatty acid-regulated transcription factors in the liver. Annu Rev Nutr.

[B73] Kalyvas A, BaskakisC, MagriotiV (2009). Differing roles for members of the phospholipase A2 superfamily in experimental autoimmune encephalomyelitis. Brain.

[B74] Kelly PA, GruberSA, Behbod F (1997). Sirolimus, a new, potent immunosuppressive agent. Pharmacotherapy.

[B75] Kern PA, Ranganathan S, Li C (2001). Adipose tissue tumor necrosis factor and interleukin-6 expression in human obesity and insulin resistance. Am J Physiol Endocrinol Metab.

[B76] Kim DH, Sarbassov DD, Ali SM (2002). mTOR interacts with raptor to form a nutrient-sensitive complex that signals to the cell growth machinery. Cell.

[B77] Kim JE, ChenJ (2004). regulation of peroxisome proliferator-activated receptor-gamma activity by mammalian target of rapamycin and amino acids in adipogenesis. Diabetes.

[B78] Kleinewietfeld M, ManzelA, TitzeJ (2013). Sodium chloride drives autoimmune disease by the induction of pathogenic TH17 cells. Nature.

[B79] Knazek RA, Liu SC (1979). Dietary essential fatty acids are required for maintenance and induction of prolactin receptors. Proc Soc Exp Biol Med.

[B80] KnowlesPF, Marsh D, Rattle HWE (1976). Magnetic resonance of biomolecules.

[B81] Kohn AD, Summers SA, Birnbaum MJ (1996). Expression of a constitutively active Akt Ser/Thr kinase in 3T3-L1 adipocytes stimulates glucose uptake and glucose transporter 4 translocation. J Biol Chem.

[B82] Kroger J, Zietemann V, Enzenbach C (2011). Erythrocyte membrane phospholipid fatty acids, desaturase activity, and dietary fatty acids in relation to risk of type 2 diabetes in the European Prospective Investigation into Cancer and Nutrition (EPIC)-Potsdam Study. Am J Clin Nutr.

[B83] Kumar S, PatelR, MooreS (2013). Estrogen receptor beta ligand therapy activates PI3K/Akt/mTOR signaling in oligodendrocytes and promotes remyelination in a mouse model of multiple sclerosis. Neurobiol Dis.

[B84] Kury PG, Ramwell PW, McConnell HM (1974). The effect of prostaglandins E1 and E2 on the human erythrocyte as monitored by spin labels. Biochem Biophys Res Commun.

[B85] Lai CS, Hopwood LE, Swartz HM (1980). Electron spin resonance studies of changes in membrane fluidity of Chinese hamster ovary cells during the cell cycle. Biochim Biophys Acta.

[B86] Lamming DW, Ye L, Katajisto P (2012). Rapamycin-induced insulin resistance is mediated by mTORC2 loss and uncoupled from longevity. Science.

[B87] Laplante M, Sabatini DM (2009). An emerging role of mTOR in lipid biosynthesis. Curr Biol.

[B88] Lee K, Gudapati P, Dragovic S (2010). Mammalian target of rapamycin protein complex 2 regulates differentiation of Th1 and Th2 cell subsets via distinct signaling pathways. Immunity.

[B89] Lerman A, McConnell JP (2008). Lipoprotein-associated phospholipase A2: a risk marker or a risk factor?. Am J Cardiol.

[B90] Lopez-ValesR, Navarro X, Shimizu T (2008). Intracellular phospholipase A(2) group IVA and group VIA play important roles in Wallerian degeneration and axon regeneration after peripheral nerve injury. Brain.

[B91] MacPhee CH, Moores KE, Boyd HF (1999). Lipoprotein-associated phospholipase A2, platelet-activating factor acetylhydrolase, generates two bioactive products during the oxidation of low-density lipoprotein: use of a novel inhibitor. Biochem J.

[B92] Malhotra A (2013). Saturated fat is not the major issue. BMJ.

[B93] Manku MS, Horrobin DF, Morse NL (1984). Essential fatty acids in the plasma phospholipids of patients with atopic eczema. Br J Dermatol.

[B94] Masuda S, Murakami M, Mitsuishi M (2005). Expression of secretory phospholipase A2 enzymes in lungs of humans with pneumonia and their potential prostaglandin-synthetic function in human lung-derived cells. Biochem J.

[B95] Mauvoisin D, Rocque G, Arfa O (2007). Role of the PI3-kinase/mTor pathway in the regulation of the stearoyl CoA desaturase (SCD1) gene expression by insulin in liver. J Cell Commun Signal.

[B96] McMurchie EJ, Raison JK (1979). Membrane lipid fluidity and its effect on the activation energy of membrane-associated enzymes. Biochim Biophys Acta.

[B97] Merkel SN, Mogilevskaja M, Mengel H, Haller A, Schwarz B (2006). Side effects of sirolimus. Transplant Proc.

[B98] Miossec P, Korn T, Kuchroo VK (2009). Interleukin-17 and type 17 helper T cells. N Engl J Med.

[B99] Miyazaki M, Ntambi JM, Vance JE (2008). Fatty acid desaturation and chain elongation in mammals. Biochemistry of lipids, lipoproteins and membranes.

[B100] Mohan CD, Srinivasa V, Rangappa S (2016). Trisubstituted-imidazoles induce apoptosis in human breast cancer cells by targeting the oncogenic PI3K/Akt/mTOR signaling pathway. PLoS One.

[B101] Monroy A, Kamath S, Chavez AO (2009). Impaired regulation of the TNF-alpha converting enzyme/tissue inhibitor of metalloproteinase 3 proteolytic system in skeletal muscle of obese type 2 diabetic patients: a new mechanism of insulin resistance in humans. Diabetologia.

[B102] Murray JT, Tee AR (2018). Mechanistic target of rapamycin (mTOR) in the cancer setting. Cancers (Basel).

[B103] Nakamura MT, Nara TY (2004). Structure, function, and dietary regulation of delta6, delta5, and delta9 desaturases. Annu Rev Nutr.

[B104] Narayanan SP, Flores AI, Wang F (2009). Akt signals through the mammalian target of rapamycin pathway to regulate CNS myelination. J Neurosci.

[B105] Nicoletti F, Di Marco R, Mangano K (2001). Increased serum levels of interleukin-18 in patients with multiple sclerosis. Neurology.

[B106] Obukowicz MG, Raz A, Pyla PD (1998). Identification and characterization of a novel delta6/delta5 fatty acid desaturase inhibitor as a potential anti-inflammatory agent. Biochem Pharmacol.

[B107] Peng T, Golub TR, Sabatini DM (2002). The immunosuppressant rapamycin mimics a starvation-like signal distinct from amino acid and glucose deprivation. Mol Cell Biol.

[B108] Pennline KJ, Roque-Gaffney E, Monahan M (1994). Recombinant human IL-10 prevents the onset of diabetes in the nonobese diabetic mouse. Clin Immunol Immunopathol.

[B109] Pike, KC, Calder PC, Inskip HM (2012). Maternal plasma phosphatidylcholine fatty acids and atopy and wheeze in the offspring at age of 6 years. Clin Dev Immunol.

[B110] Pilon M (2016). Revisiting the membrane-centric view of diabetes. Lipids Health Dis.

[B111] Porstmann T, Santos CR, Griffiths B (2008). SREBP activity is regulated by mTORC1 and contributes to Akt-dependent cell growth. Cell Metab.

[B112] Qi L, Sun K, Zhuang Y (2017). Study on the association between PI3K/AKT/mTOR signaling pathway gene polymorphism and susceptibility to gastric cancer. J BUON.

[B113] Quach ND, Arnold RD, Cummings BS (2014). Secretory phospholipase A2 enzymes as pharmacological targets for treatment of disease. Biochem Pharmacol.

[B114] Quinn PJ, Joo F, Vigh L (1989). The role of unsaturated lipids in membrane structure and stability. Prog Biophys Mol Biol.

[B115] Rabini RA, Galassi R, Staffolani R (1993). Alterations in Na+/K(+)-ATPase activity and fluidity of erythrocyte membranes from relatives of insulin dependent diabetic patients. Diabetes Res.

[B116] Rapoport MJ, Jaramillo A, Zipris D (1993). Interleukin 4 reverses T cell proliferative unresponsiveness and prevents the onset of diabetes in nonobese diabetic mice. J Exp Med.

[B117] Reddy BS (1994). Chemoprevention of colon cancer by dietary fatty acids. Cancer Metastasis Rev.

[B118] Redig AJ, VakanaE, PlataniasLC (2011). Regulation of mammalian target of rapamycin and mitogen activated protein kinase pathways by BCR-ABL. Leuk Lymphoma.

[B119] Rezapour-Firouzi S, Watson R (2017). Herbal oil supplement with hot-nature diet for multiple sclerosis. Nutrition and Lifestyle in Neurological Autoimmune Diseases.

[B120] Rezapour-Firouzi S, Arefhosseini SR, Ebrahimi-Mamaghani M (2015). Alteration of delta-6-desaturase (FADS2), secretory phospholipase-A2 (sPLA2) enzymes by Hot-nature diet with co-supplemented hemp seed, evening primrose oils intervention in multiple sclerosis patients. Complement Ther Med.

[B121] Rezapour-Firouzi S, Arefhosseini SR, Baradaran B (2013a). Erythrocyte membrane fatty acids in multiple sclerosis patients and hot-nature dietary intervention withco-supplemented hemp-seed and evening-primrose oils. Afr J Tradit Complement Altern Med.

[B122] Rezapour-Firouzi S, Arefhosseini SR, Baradaran B (2013b). Association of expanded disability status scale and cytokines after intervention with co-supplemented hemp seed, evening primrose oils and hot-natured diet in multiple sclerosis patients. Bioimpacts.

[B123] Rezapour-Firouzi S, Arefhosseini SR, Baradaran B (2013c). Immunomodulatory and therapeutic effects of Hot-nature diet and co-supplemented hemp seed, evening primrose oils intervention in multiple sclerosis patients. Complement Ther Med.

[B124] Rezapour-Firouzi S, Arefhosseini SR, Ebrahimi-Mamaghani M (2013d). Regulation of lipid-dependent membrane enzymes by hot nature diet with co-supplemented Hemp seed, evening primrose oils intervention in multiple sclerosis patients. J Pure Appl Microbio.

[B125] Rivers JP, Frankel TL (1981). Essential fatty acid deficiency. Br Med Bull.

[B126] Roncone M, Bartlett H, Eperjesi F (2010). Essential fatty acids for dry eye: A review. Cont Lens Anterior Eye.

[B127] Ruzicka T, Simmet T, Peskar BA (1986). Skin levels of arachidonic acid-derived inflammatory mediators and histamine in atopic dermatitis and psoriasis. J Invest Dermatol.

[B128] Sakaguchi S (2004). Naturally arising CD4+ regulatory t cells for immunologic self-tolerance and negative control of immune responses. Annu Rev Immunol.

[B129] Salvati S, Attorri L, Avellino C (2000). Diet, lipids and brain development. Dev Neurosci.

[B130] Samuel VT, Shulman GL (2012). Mechanisms for insulin resistance: common threads and missing links. Cell.

[B131] Samuels Y, Wang Z, Bardelli A (2004). High frequency of mutations of the PIK3CA gene in human cancers. Science.

[B132] Sarbassov DD, Guertin DA, Ali SM (2005). Phosphorylation and regulation of Akt/PKB by the rictor-mTOR complex. Science.

[B133] Schonrock LM, Gawlowski G, Bruck W (2000). Interleukin-6 expression in human multiple sclerosis lesions. Neurosci Lett.

[B134] Schroeder F, Perlmutter JF, Glaser M (1976). Isolation and characterization of subcellular membranes with altered phospholipid composition from cultured fibroblasts. J Biol Chem.

[B135] Scott B, Liblau R, Degermann S (1994). A role for non-MHC genetic polymorphism in susceptibility to spontaneous autoimmunity. Immunity.

[B136] Senn JJ, Klover PJ, Nowak IA (2003). Suppressor of cytokine signaling-3 (SOCS-3), a potential mediator of interleukin-6-dependent insulin resistance in hepatocytes. J Biol Chem.

[B137] Shinitzky M, M. Shinitzky (1984). Membrane fluidity and cellular functions. Physiology of membrane Fluidity.

[B138] Shinitzky M, Barenholz Y (1978). Fluidity parameters of lipid regions determined by fluorescence polarization. Biochim Biophys Acta.

[B139] Sternberg Z, Drake A, Sternberg DS (2012). Lp-PLA2: inflammatory biomarker of vascular risk in multiple sclerosis. J Clin Immunol.

[B140] Stockard JE, SasteMD, BenfordVJ (2000). Effect of docosahexaenoic acid content of maternal diet on auditory brainstem conduction times in rat pups. Dev Neurosci.

[B141] Sul HS, SmithS, Vance JE, Vance D (2008). Fatty acid synthesis in eukaryotes. Biochemistry of lipids, lipoproteins and membranes.

[B142] Sun GY, Xu J, Jensen MD (2004). Phospholipase A2 in the central nervous system: implications for neurodegenerative diseases. J Lipid Res.

[B143] Tong P, Thomas T, Berrish T (1995). Cell membrane dynamics and insulin resistance in non-insulin-dependent diabetes mellitus. Lancet.

[B144] Tremblay F, Marette A (2001). Amino acid and insulin signaling via the mTOR/p70 S6 kinase pathway A negative feedback mechanism leading to insulin resistance in skeletal muscle cells. J Biol Chem.

[B145] Trembleau S, Penna G, Bosi E (1995). Interleukin 12 administration induces T helper type 1 cells and accelerates autoimmune diabetes in NOD mice. J Exp Med.

[B146] Turnbaugh PJ, Ley RE, Mahowald MA (2006). An obesity-associated gut microbiome with increased capacity for energy harvest. Nature.

[B147] Ueki K, Yamamoto-Honda R, Kaburagi Y (1998). Potential role of protein kinase B in insulin-induced glucose transport, glycogen synthesis, and protein synthesis. J Biol Chem.

[B148] Vessby B (2003). Dietary fat, fatty acid composition in plasma and the metabolic syndrome. Curr Opin Lipidol.

[B149] Vittos O, ToanaB, VittosA (2012). Lipoprotein-associated phospholipase A2 (Lp-PLA2): a review of its role and significance as a cardiovascular biomarker. Biomarkers.

[B150] Waczulikova I, SikurovaL, CarskyJ (2000). Decreased fluidity of isolated erythrocyte membranes in type 1 and type 2 diabetes The effect of resorcylidene aminoguanidine. Gen Physiol Biophys.

[B151] Wang Q, SomwarR, BilanPJ (1999). Protein kinase B/Akt participates in GLUT4 translocation by insulin in L6 myoblasts. Mol Cell Biol.

[B152] Wang X, WangW, Xu J (2013). Effect of rapamycin and interleukin-2 on regulatory CD4+CD25+Foxp3+ T cells in mice after allogenic corneal transplantation. Transplant Proc.

[B153] Warensjo E, RiserusU, VessbyB (2005). Fatty acid composition of serum lipids predicts the development of the metabolic syndrome in men. Diabetologia.

[B154] Weijers RN (2012). Lipid composition of cell membranes and its relevance in type 2 diabetes mellitus. Curr Diabetes Rev.

[B155] Wieman HL, Wofford JA, Rathmell JC (2007). Cytokine stimulation promotes glucose uptake via phosphatidylinositol-3 kinase/Akt regulation of Glut1 activity and trafficking. Mol Biol Cell.

[B156] Wu C, YosefN, ThalhamerT (2013). Induction of pathogenic TH17 cells by inducible salt-sensing kinase SGK1. Nature.

[B157] Yasuda M, Tanaka Y, Kume S (2014). Fatty acids are novel nutrient factors to regulate mTORC1 lysosomal localization and apoptosis in podocytes. Biochim Biophys Acta.

[B158] Yedgar S, CohenY, Shoseyov D (2006). Control of phospholipase A2 activities for the treatment of inflammatory conditions. Biochim Biophys Acta.

[B159] Yehuda S, Simopoulos AP, Cleland LG (2003). Omega-6/omega-3 ratio and brain related functions. Omega-6/omega-3 essential fatty acid ratio: the scientific evidence.

[B160] Yehuda S, RabinovitzS, CarassoRL (2000). Fatty acid mixture counters stress changes in cortisol, cholesterol, and impair learning. Int J Neurosci.

[B161] Yehuda S, Rabinovitz S, Mostofsky DI (2005). Essential fatty acids and the brain: from infancy to aging. Neurobiol Aging.

[B162] Yu G, Bjorksten B (1998). Polyunsaturated fatty acids in school children in relation to allergy and serum IgE levels. Pediatr Allergy Immunol.

[B163] Yuan LF, Li GD, Ren XJ (2015). Rapamycin ameliorates experimental autoimmune uveoretinitis by inhibiting Th1/Th2/Th17 cells and upregulating CD4+CD25+ Foxp3 regulatory T cells. Int J Ophthalmol.

[B164] Yuan M, PinoE, WuL (2012). Identification of Akt-independent regulation of hepatic lipogenesis by mammalian target of rapamycin (mTOR) complex 2. J Biol Chem.

[B165] Zhang HH, HuangJ, DuvelK (2009). Insulin stimulates adipogenesis through the Akt-TSC2-mTORC1 pathway. PLoS One.

[B166] Zheng Y, CollinsSL, LutzMA (2007). A role for mammalian target of rapamycin in regulating T cell activation versus anergy. J Immunol.

[B167] Zivkovic AM, GermanJB, LebrillaCB (2011). Human milk glycobiome and its impact on the infant gastrointestinal microbiota. Proc Natl Acad Sci U S A.

[B168] Zoncu R, Efeyan A, Sabatini DM (2011). mTOR: from growth signal integration to cancer, diabetes and ageing. Nat Rev Mol Cell Biol.

